# What is the Cause of This Patient’s Symptoms?

**Published:** 2012-03-01

**Authors:** Mary Petrofsky, Laura Zitella

**Affiliations:** From Stanford Hospital and Clinics, Stanford, California

## Abstract

**Figure 1:**
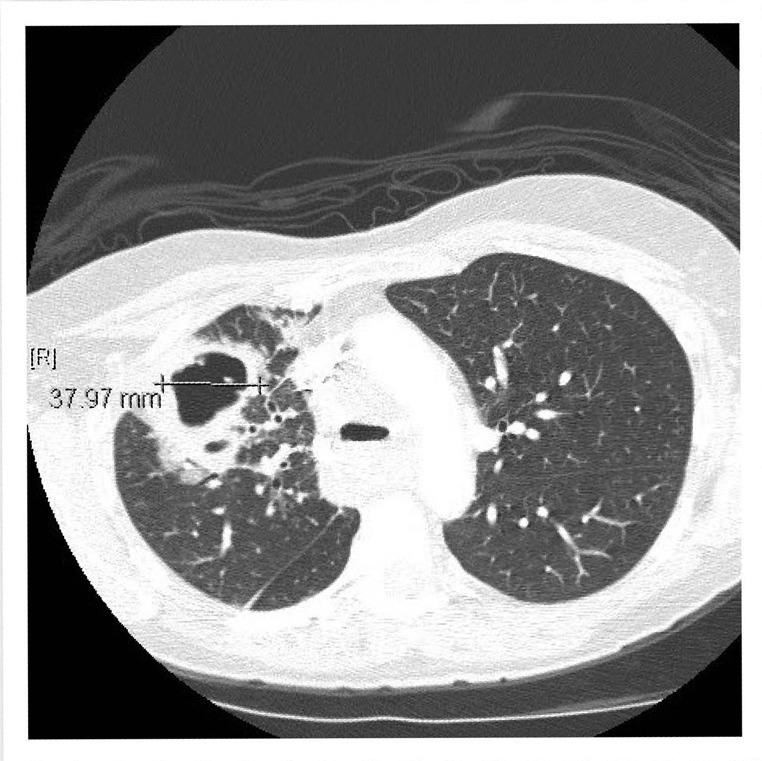

## History

Ms. C., a 53-year-old nonsmoking woman, presented with a productive cough, dyspnea, and orthopnea. She was sequentially treated with antibiotics, allergy medications, and steroids without symptom improvement. Chest x-ray revealed a widened mediastinum and right upper lobe (RUL) lesion. A CT of the thorax showed a 1.5 × 1.3 cm RUL lesion with slight cavitation and a 7-cm mediastinal mass causing 50% constriction of the right mainstem bronchus.

Ms. C. became profoundly hypoxic and was unable to undergo a bronchoscopic biopsy. She was transferred to a tertiary care medical center where endobronchial ultrasound-guided biopsy of the mediastinal mass was performed; pathology demonstrated squamous cell carcinoma. Staging PET-CT done 9 days after the initial CT showed an increase in the size of the RUL cavitary lesion to 2.8 × 2.2 cm and stable size of the mediastinal mass with no distant metastases. These findings were consistent with stage IIIB squamous cell lung carcinoma.

Due to Ms. C.’s poor performance status, she began treatment with weekly carboplatin/paclitaxel chemotherapy and concurrent radiation therapy. Two weeks after the start of chemoradiation, her hypoxia and performance status improved sufficiently to allow her discharge from the hospital. She continued the radiation treatment as an outpatient, and her improved performance status permitted a change in the concurrent chemotherapy to the more aggressive chemotherapy regimen of cisplatin and etoposide administered every 3 weeks.

## Chief Complaint

On day 10 of the first cycle of outpatient cisplatin/etoposide therapy (4 weeks after the staging PET-CT), Ms. C. presented to the emergency department with fevers, hemoptysis up to 1 tablespoon, right subscapular pain rated 4/10, and progressive dyspnea.

## Physical Examination and Diagnostic Studies

On physical examination, vital signs were temperature 38.5°C, heart rate 110, respirations 24, blood pressure 110/62, and O_2_ saturation 96% on 2 L/min O_2_. There were diminished breath sounds in the RUL and occasional crackles bilaterally. Clinically significant laboratory values included low blood counts: white blood cells 1.6, hemoglobin 9.4, hematocrit 27.0, platelets 120, and absolute neutrophil count 720, consistent with recent chemotherapy. A CT of the thorax (Figure 1) showed a marked interval increase in the size of the RUL cavitary lesion that measured 3.5 × 4.5 cm and decreased size of the mediastinal nodal mass.

## Choose the correct diagnosis:

- A: TUMOR PROGRESSION
- B: FUNGAL INFECTION
- C: TUBERCULOSIS

## Scroll down for correct answer.

**Figure 1:**
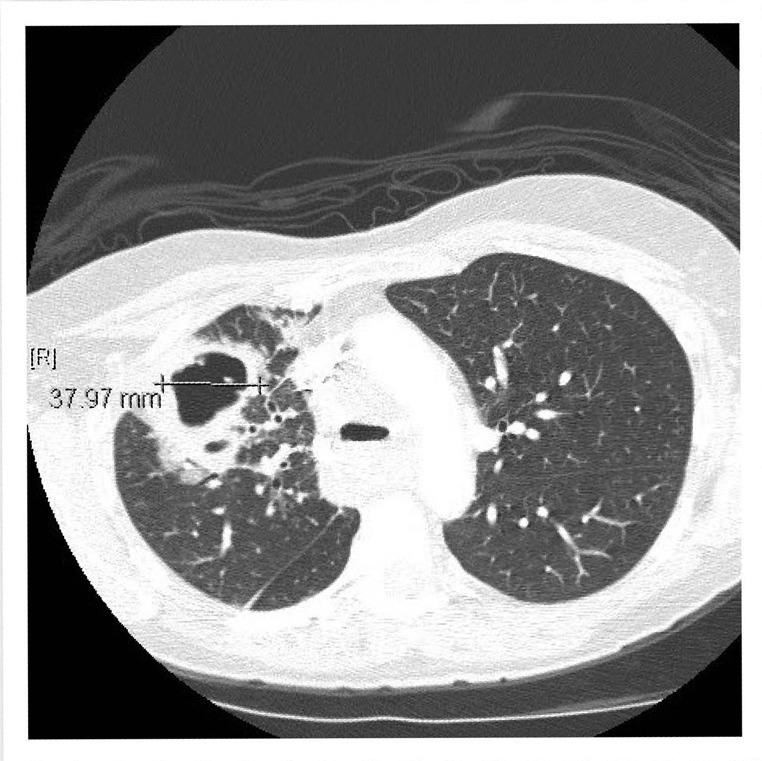


## Correct Answer

***Fungal infection (aspergillosis).*** A discordant tumor response to chemoradiation with rapid expansion of the cavitary portion of the RUL lesion and decreased size of the mediastinal lesion is uncommon. The presence of fevers, dyspnea, and hemoptysis warranted an infectious disease evaluation. Serology tests for aspergillosis, tuberculosis, coccidioidomycosis, cryptococcosis, histoplasmosis, brucellosis, and blastomycosis were negative. Serum quantiferon assay was negative. Aerobic, anaerobic, and fungal blood cultures showed no growth. Sputum AFB (acid-fast bacilli) and DFA (direct-fluorescent antibody) were negative. Since the noninvasive tests were nondiagnostic, biopsy of the RUL lesion was performed to definitively differentiate between tumor progression and infection. Pathology from a CT-guided fine-needle aspiration of the RUL lesion showed extensive necrosis with branching hyphae, which was morphologically consistent with aspergillosis (Hope, Walsh, & Denning, 2005). Three weeks after the biopsy, fungal cultures from the biopsy grew *Aspergillus fumigatus*.

Aspergillosis is caused by *Aspergillus* species. Humans routinely inhale fungal spores. Patients who are immunocompromised are at risk for invasive aspergillosis (IA), which occurs when the fungus grows and invades surrounding tissue layers, often leading to vascular invasion with hemoptysis (Kim, 2010).

Invasive aspergillosis is rapidly progressive and often fatal in immunocompromised patients (Walsh et al., 2008). Typical symptoms include cough, hemoptysis, dyspnea, chest pain, and fever (Kim, 2010). Diagnosis is based on a combination of serology tests, radiographic evidence, and pathology review (Fricke et al., 2011). Fungal cultures are the most specific diagnostic tool, but they are insensitive and slow to be resulted. Radiography may reveal classic signs of *Aspergillus* infection: a solitary nodule with a halo sign (an early sign consisting of a zone of low attenuation surrounding the lesion), consolidation with cavitation, and/or the air crescent sign (a crescenteric and radiolucent area within an area of consolidation or nodularity), but these findings are not always present (Kuhlman et al., 1985). Serum galactomannan and 1-3-β-D-glucan assays detect components of the *Aspergillus* cell wall and, with radiologic findings, suggest invasive aspergillosis (Walsh et al., 2008). Early detection is critical to successful treatment. New real-time PCR tests that may provide results from serum and tissue samples in 4 to 6 hours (Fricke et al., 2011) are in development.

## Explanation of Incorrect Answers

**Tumor progression** is less likely in the setting of fevers, rapid growth of the RUL lesion, and radiologic evidence of treatment response of the mediastinal lesion with concurrent chemoradiation. The area of the RUL lesion that enlarged was primarily the cavity. During radiation therapy, necrotic areas of a tumor may expand as a result of tumor necrosis and inflammation, creating a cavitary lesion (Frytak et al., 1988), but this would indicate tumor response, not tumor progression.

**Tuberculosis (TB)** is an infection caused by *Mycobacterium tuberculosis*. Infections can be latent or active, and patients with latent TB who undergo chemotherapy have a risk of developing active TB. Symptoms of active pulmonary TB include cough, weight loss, fatigue, fever, and night sweats. Hemoptysis and chest pain are later signs of TB (Mayo Clinic, 2011). Common radiographic findings are hilar lymphadenopathy, pleural effusions, and pulmonary infiltrates (Choyke et al., 1983).

## Management

Antifungal therapy should be started early, during the diagnostic workup, in patients strongly suspected to have IA. Voriconazole and deoxycholate amphotericin B are approved by the FDA for treatment of primary IA. Voriconazole is the drug of choice due to the superior effectiveness and toxicity profile compared to conventional amphotericin B (Herbrecht et al., 2002; Walsh et al., 2008). However, the liposomal formulation of amphotericin B is less toxic than conventional amphotericin B. Due to the early diagnostic uncertainty, Ms. C. was initially treated with broad-spectrum antimicrobial therapy, including voriconazole, but therapy was narrowed to voriconazole after hyphae were seen in the biopsy. Due to continued fevers, Ms. C. remained on IV voriconazole for 3 weeks prior to switching to oral therapy. Chemotherapy was held. In oncology patients, antifungal therapy should be continued until the end of immunosuppression and until the lesions have resolved (Walsh et al., 2008).
